# Residual Longevity of Recaptured Sterile Mosquitoes as a Tool to Understand Field Performance and Reveal Quality

**DOI:** 10.3390/insects15110826

**Published:** 2024-10-23

**Authors:** Georgios Balatsos, Laura Blanco-Sierra, Vasileios Karras, Arianna Puggioli, Hugo Costa Osório, Romeo Bellini, Dimitrios P. Papachristos, Jérémy Bouyer, Frederic Bartumeus, Nikos T. Papadopoulos, Antonios Michaelakis

**Affiliations:** 1 Scientific Directorate of Entomology and Agricultural Zoology, Benaki Phytopathological Institute, 14561 Kifissia, Greece; g.balatsos@bpi.gr (G.B.); v.karras@bpi.gr (V.K.); d.papachristos@bpi.gr (D.P.P.); 2 Centre d’Estudis Avançats de Blanes (CEAB-CSIC), 14, 17300 Blanes, Girona, Spain; lblanco@ceab.csic.es (L.B.-S.); fbartu@ceab.csic.es (F.B.); 3 Centro Agricoltura Ambiente “G. Nicoli”, 40014 Crevalcore, Italy; apuggioli@caa.it (A.P.); rbellini@caa.it (R.B.); 4 Centre for Vectors and Infectious Diseases Research Doutor Francisco Cambournac (CEVDI), National Institute of Health Doutor Ricardo Jorge (INSA), Avenida da Liberdade 5, 2965-575 Palmela, Portugal; hugo.osorio@insa.min-saude.pt; 5 Faculty of Medicine, Environmental Health Institute (ISAMB), University of Lisbon, Av. Prof. Egas Moniz, Ed. Egas Moniz, Piso 0, Ala C, 1649-028 Lisboa, Portugal; 6 ASTRE, CIRAD, INRAE, Plate Forme CYROI, 2 rue Maxime Rivière, 97491 Sainte-Clotilde, La Réunion, France; jeremy.bouyer@cirad.fr; 7 Institució Catalana de Recerca i Estudis Avançats (ICREA), Passeig de Lluís Companys 23, 08010 Barcelona, Spain; 8 Centre de Recerca Ecològica i Aplicacions Forestals (CREAF), Cerdanyola del Vallès, 08193 Barcelona, Spain; 9 Department of Agriculture, Crop Production and Rural Environment, University of Thessaly, 38446 Magnisias, Greece

**Keywords:** *Aedes albopictus*, sterile insect technique, mosquito longevity, captive cohort method, vector control

## Abstract

Invasive mosquito species, such as *Aedes albopictus*, pose significant threats to both ecosystems and public health due to their role in transmitting diseases, such as dengue, Zika, and chikungunya. The Sterile Insect Technique (SIT) is a promising vector control strategy aimed at reducing mosquito populations by releasing sterile males to mate with wild females and reduce their reproduction rates. In this study, we employed the captive cohort method, which assesses the remaining longevity of randomly caught released individuals, to assess the longevity and frailty dynamics of sterile and non-sterile *Ae. albopictus* males. Using a mark–release–recapture approach (MRR), we compared the residual lifespan of sterile and non-sterile released males with that of wild, non-sterile males, aiming to understand the frailty dynamics of released males and, therefore, their quality and field performance. Contrary to expectations, our results revealed that released sterile males showed increased longevity compared to non-sterile males. Further, the marking process did not impact the longevity between lab-kept and marked males, suggesting that the marking process does not adversely affect survival under controlled conditions. These findings underscore the importance of optimizing pre-release and mass-rearing practices to enhance the effectiveness of SIT programs. Our study also demonstrates for the first time the use of the captive cohort method for understanding the biological dynamics of sterile mosquito populations in SIT programs, providing valuable insights for improving vector control strategies.

## Simple Summary

The current study focused on understanding the longevity and frailty of sterile, non-sterile, and wild male mosquitoes subjected to different treatments, using the novel captive cohort method. Key findings include that marking mosquitoes, following IAEA protocols, had an insignificant effect on longevity under controlled conditions, and that sterilization had no negative effect on male longevity. Moreover, we recorded that exposure to the wild increased post-capture longevity, particularly for sterile males, with longer time in the wild correlating with extended lifespan. Interestingly, the wild experience seems to benefit sterile males more than non-sterile ones, possibly due to demographic selection or hormetic effects. This suggests a possible advantage in field performance for sterile males, which is a key consideration for SIT programs. These findings underscore the importance of ongoing research to optimize rearing, sterilization, and transportation methods for sterile males, ultimately enhancing their performance and longevity in field applications.

## 1. Introduction

Invasive mosquito species (IMS) pose a substantial threat to ecosystems, native mosquito populations, and diversity, and more importantly, to human health, since many of them can transmit numerous diseases, including dengue virus, Zika virus, chikungunya virus, and Japanese encephalitis virus. The IMS *Aedes albopictus* is currently recognized as one of the most aggressive invaders in many areas all over the globe, with major epidemiological importance since it vectors some of the aforementioned diseases [1,2,3,4]. Efforts to control *Aedes* spp., including *Ae. albopictus* populations, through integrated vector management are essential for reducing the spread of these diseases; thus, the development of novel control methods plays a critical role in mitigating the impact on human health [5,6,7]. The Sterile Insect Technique (SIT) is an eco-friendly approach to achieve insect vector management targeting eradication, prevention, and suppression. SIT involves several important steps, such as mass rearing of the target mosquito species, sex sorting, sterilization, and the release of sterile males into the target area [8,9,10,11,12]. Sterile males mate with wild females and induce sterility in wild populations, which ultimately leads to a reduction in the reproduction rate of the wild population. Therefore, the consistent and continuous release of an adequate number of competitive sterile males to wild counterparts throughout the entomological season can significantly suppress or even eliminate the wild mosquito population in the target area. The effectiveness of SIT can be enhanced with further improvements and can be effectively integrated with other control strategies, including source reduction, such as implementation of a door-to-door campaign [13,14]. Because the quality and field performance of sterile males are crucial for the success of SIT, standardized quality control (QC) tests have been developed for many insect species to which SIT has been applied. These QC tests assess adult emergence rates, flight ability, stress resistance, longevity, and ultimately mating competitiveness [14,15,16].

In Greece, the first pilot application of SIT against *Ae. albopictus* was implemented in 2018. By 2019, SIT was successfully implemented in the pilot site throughout the entire entomological season, leading to significant suppression of the local population [10,11]. In September 2020, we implemented mark–release–recapture (MRR) experiments, which are widely used across various species for estimating mosquito longevity and dispersion. In the context of SIT, mosquito longevity refers to the lifespan of sterile mosquitoes after they are released into the wild. Longevity of sterile mosquitoes in the wild is a prerequisite for their performance and is directly linked to the effectiveness of SIT. Therefore, monitoring and understanding the longevity of sterile mosquitoes is crucial for optimizing the timing and frequency of release to maximize the suppression of the mosquito population [13,15,17]. 

Gaining insights into biology, age structure, and longevity in the wild for mosquito species and other insects is challenging [18]. Release–recapture studies are often considered to infer field longevity and the “disappearance” rate after releasing is interpreted as mortality. Several methods have been considered to gain insight into the age of animals in the wild, including the reproductive age of wild-caught females, infrared spectroscopy, and cuticular hydrocarbon analysis. However, most are rather expensive and do not provide satisfactory estimates [18]. The captive cohort method, based on Carey’s Equality [19], has been proposed to estimate the age structure of wild populations. Briefly, randomly sampling individuals of unknown age from the wild that are followed to death provide vital information for the age structure of wild populations [20,21,22]. The so-called “captive cohort method” can provide information on the frailty (i.e., weak or more vulnerable to death) dynamics in wild field populations of mosquitoes, and considers the tight relationship between age, frailty, and longevity. Indeed, the captive cohort method is particularly relevant for studying mosquito populations as the remaining lifespan of captured individuals reflects both their physiological and chronological age, offering valuable information about their overall longevity [18,23].

The objective of the current study was to determine if the captive cohort method is a valuable tool to gain insights into the field biology of sterile released *Ae. albopictus* males. By comparing the residual lifespan of sterile and non-sterile released males with that of wild, non-sterile males, we aimed to understand the frailty dynamics of released males, and therefore their quality and field performance. To achieve this objective, we conducted a mark–release–recapture study (MRR), collecting live males and taking them to the laboratory, where their lifespan was determined. Both sterile and non-sterile males were released, and wild males were captured as well. Control mosquito males emerging from laboratory colonies of the different categories of mosquitos mentioned above were included in our trials as well.

## 2. Materials and Methods

### 2.1. Study Site and Mosquito Populations

Mark–release–recapture (MRR) was implemented in Vravrona, a locality in the municipality of Markopoulos, located in the Attica region of Greece, east of Athens. The area has been used for pilot tests of SIT against *Ae. albopictus* since 2018. *Aedes albopictus* eggs, collected from Vravrona, Greece, were used for rearing. Rearing was conducted in two facilities: a mass production facility in Italy and a small-scale production setup in Greece, resulting in the formation of three experimental mosquito groups: (1) sterile males produced in Italy and subsequently transported to Greece, (2) non-sterile males produced in Italy and transported to Greece, and (3) non-sterile males reared locally in Greece under small-scale production conditions. In addition to Vravrona, wild mosquitoes were collected in the semiurban area of Artemida, which has been used as a control in previous SIT studies and is located 1 km east of Vravrona [9,10,11].

### 2.2. Mass Rearing, Sterilization, and Transportation

Mass rearing was conducted at the Centro Agricoltura Ambiente (CAA) ‘G. Nicoli’, Italy, using the strain of *Ae. albopictus* that originated from this study area (Greek stain). Following sex sorting at the pupal stage, male pupae were divided into two batches. In the first batch, a subset of male pupae was sterilized via gamma radiation at a dose of 35 Gy, whereas the second batch remained non-sterile (Table 1). Sterile and non-sterile adult males were then chilled and transferred into small cylindrical plastic containers (5 cm in diameter and 5 cm in height, with an 80 cc capacity), which were labeled according to the status of the males (sterile or non-sterile). These containers were sealed with tape and placed inside a larger plastic container (PP plastic, 20 × 15 × 6 h cm, 1800 cc capacity). The sterile and non-sterile males were then transported from Italy (CAA) to Greece (Benaki Phytopathological Institute (BPI)) in a polystyrene container with an adequate quantity of phase-changing materials (PCMs) to maintain a temperature of approximately 12 °C and were delivered by express courier service from the mass production facility (CAA), as described in Mastronikolos et al. [14]. 

In the small-scale production in the insectary of BPI, *Ae. albopictus* eggs were hatched, and the larvae were reared under controlled environmental conditions (25 ± 2 °C, 65 ± 5% RH, 14:10 D:L), including a 30-min sunrise and sunset simulation. The rearing continued until the pupal stage, where sex sorting was performed. The emerged males were subsequently counted and divided into two treatments regarding their marking status (Control 1: marked and Control 2: non-marked; Table 1).

### 2.3. Marking, Mosquito Release, and Recapture 

For MRR implementation, the *Ae. albopictus* male mosquitoes were categorized based on their marking status (marked vs. unmarked), sterilization status (sterile vs. non-sterile), release status (released vs. non-released), and production origin (CAA—mass production vs. BPI—small-scale production) (Table 1). Marking was performed using a fluorescent dye, and the impact of marking on mosquito survival was assessed by comparing the survival rates of marked and unmarked individuals to differentiate between the experimental groups. Specifically, the sterile male mosquitoes were dusted according to the MRR protocol of the International Atomic Energy Agency (IAEA) [17], with 5 mg of dust per 1000 adult males. The sterile males were released at a single central point, according to the protocol described by Balatsos et al. (2024) [11]. Two releases were conducted on 11 September and 25 September 2020. The first release consisted of 25,000 male mosquitoes (12,500 sterile and 12,500 non-sterile males). The second release involved 31,000 male mosquitoes (15,500 sterile and 15,500 non-sterile). The human landing collection (HLC) technique was used to recapture males alive from the field (from 3 to 7 and 20 to 25 days after each release), employing a manual battery aspirator for 5 min at each sampling station (established within a radius of 50 m from the release point). Recaptured males were immediately transferred alive to a laboratory insectary (25 °C, 65% RH, 14:10 L) at the BPI premises. In the insectary, the males were sorted by color to indicate their status (sterile, non-sterile, or wild), placed individually in 400 mL transparent plastic cages with a side opening (35 cm^2^), covered with muslin, and provided with a 10% sugar solution, as described by Papadopoulos et al. (2016) [18]. Their remaining lifespans were monitored daily. Depending on the recapture rates, as many captured males as possible were taken to the laboratory and assigned to residual longevity tests. 

### 2.4. Data Analysis

We explored the impacts of marking, sterilization, release, and days spent outside on the longevity of males from two different production methods (CAA and BPI, representing mass and small-scale production, respectively) using the same strain (Vravrona population) (Table 1). Given the non-normal distribution of the data, we employed Wilcoxon Mann–Whitney and Kruskal–Wallis tests to assess differences in survival rates [24]. To evaluate the effects of marking, sterilization, and release on longevity, the Kaplan–Meier method to estimate survival functions for each variable was considered. This analysis was conducted separately for males from both laboratories (Kaplan and Meier, 1958) [24]. Censored observations (*n* = 33) encompassed both adult mosquitoes that escaped and individuals who died in an artifactual manner (e.g., due to human-induced causes). We performed a Cox regression analysis to assess the effect of the different processes on the longevity of males. Hazard ratios (HRs) and 95% confidence intervals (95% CIs) were calculated to determine the impact and significance of each of the previously mentioned variables. We separated the database into two categories according to production origin of the males, with the aim of studying the longevity of males from Greece (not sterile and not transported) and the males from Italy (sterile/non-sterile and transported) separately. Males captured from the wild in Greece were excluded from the Cox analysis to simplify the interpretation of the results. Potential correlation and collinearity were assessed using the corrplot and car packages for R version 4.2.0, respectively. Survival analysis and model validation were implemented using the survival and survminer packages in R [25,26,27,28,29].

## 3. Results

### 3.1. Effect of Marking on Longevity

**Figure 1:**
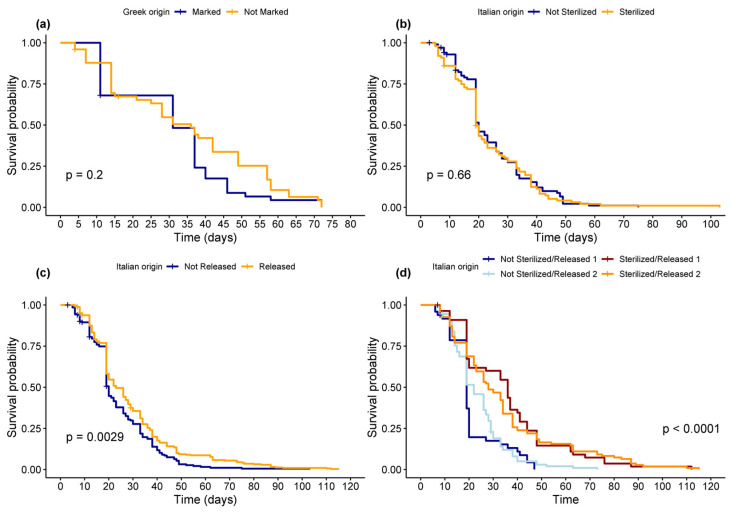
Kaplan–Meier curves of cumulative survival (**a**) of males marked vs. not-marked (small-scale production, BPI); (**b**) sterile vs. not-sterile, transported and kept in the laboratory; (**c**) released vs. non-released and transported; (**d**) sterile vs. non-sterile, transported and released. “Transported” males were produced in a mass production facility (CAA, Italy) and transported to Greece (BPI). The *p* values of the long-rank test are given on each graph.

Considering males from the small-scale production in Greece, we tested the effect of marking on adult longevity. As shown in Table 2, the average lifespan of marked and non-marked males that emerged in the laboratory was similar. A comparison of Kaplan–Meier survival curves followed by a long-rank test confirmed this finding (Figure 1a).

### 3.2. Effect of Sterilization

The average lifespan of sterile and non-sterile males received from CAA, which emerged in BPI and were kept in the laboratory, was similar (Table 3; Wilcoxon Mann–Whitney test, *p* < 0.05; Figure 1; *p* = 0.66).

### 3.3. Effect of Release in the Wild 

The overall longevity of males received from CAA, Italy (Table 1), and released in the wild, regardless of sterilization, was longer that of males that never experienced the wild (kept under laboratory conditions; Figure 1c, *p* = 0.0029).

### 3.4. Effect of Production Origin and Transportation 

The average lifespan of non-sterile males obtained from the small-scale rearing at BPI was 33.44 ± 20.83 SD, which was significantly longer than that of non-sterile males transported from Italy to BPI, with an average lifespan of 23.25 ± 13.16 SD (Table 2; Wilcoxon Mann–Whitney test, W = 29,386, *p* < 0.05) 

### 3.5. Effect of Sterilization and Release Status on Longevity

The proportion of recaptured individuals was very low for both sterile and non-sterile individuals (0.58 and 0.56%, respectively). A Cox regression model with interactions was used to test the effects of sterilization (sterile vs. non-sterile), experience in the wild (released vs. non-released), and the number of days spent in the wild. The results revealed no difference between the two release groups (Table 4) and indicated that released males lived longer after being caught and transferred to the laboratory compared to those that never experienced the wild (Table 5). Likewise, longevity of sterile males was longer than that of non-sterile males, regardless of whether they experienced the wild or not. Additionally, the longer the time spent in the wild, the greater the longevity observed in the laboratory. Interestingly, the significant interaction between release and sterilization demonstrates that sterile males gained a longevity advantage over non-sterile males after being released into the wild.

### 3.6. Effect of Production Origin on Longevity

We separated the database into two categories regarding the production origin of males, with the aim of studying the longevity of males from Greece (not sterile and not transported) and the males from the mass production facility in Italy (sterile/non-sterile and transported) separately. Results from the Cox analysis revealed slight differences in longevity between laboratory-derived males from both production facilities (excluding males captured in the wild in Greece), with mosquitoes from Greece exhibiting the longest longevity (W = 29,386, *p* < 0.05).

### 3.7. Longevity of Wild Males

**Figure 2:**
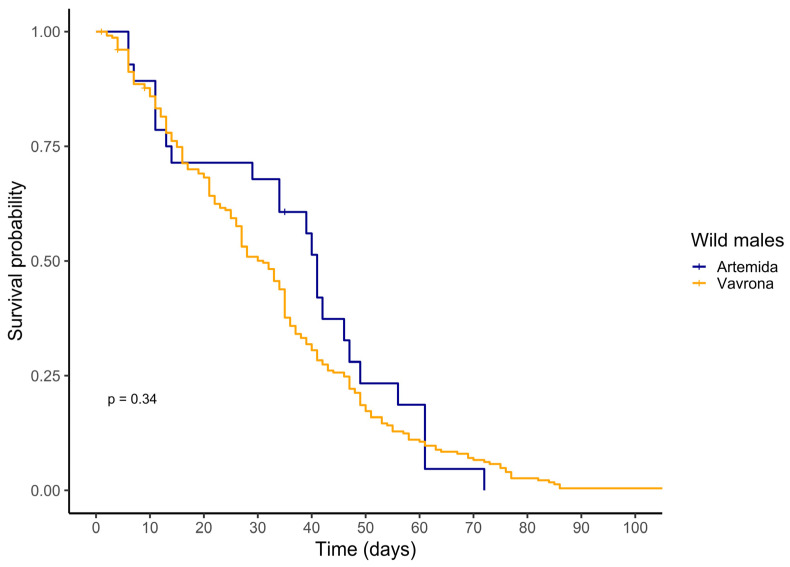
Kaplan–Meier curves of cumulative survival for wild males captured in the sterile male release area (Vravrona) and the nearby control area (Artemida). The *p*-value of the long-rank test is provided on the graph.

Similarly, the mean post-capture longevity of males captured in the control locality of Artemida and in Vravrona, the treated area where SIT males had been previously released, was similar (Table 6). Likewise, the captive survival curves followed a similar pattern (Figure 2).

## 4. Discussion

Focusing on the longevity and frailty of sterile, non-sterile, and wild male mosquitoes subjected to different treatments, and adopting the novel captive cohort method, the current study revealed that: (a) marking following the standard protocol of the IAEA had negligible effects on longevity under constant laboratory conditions, (b) likewise, sterilization did not affect male longevity, and (c) mass rearing and/or transportation seemed to induce stress and reduce longevity. The experience of the wild (released males) (d) increased male longevity, and the longer the exposure to the wild, the longer the lifespan. The wild experience (e) seemed to elicit a differential effect on post-capture male lifespan between sterile and non-sterile males, with a clear advantage for sterile males. Understanding the frailty dynamics of released sterile males serves as a proxy for their field performance and quality, complementing MRR data regarding the disappearance rate of released males. Indeed, our data provide evidence for clear differentiation among the different cohorts of recaptured individuals regarding captive lifespan (Table 4 and Table 5). Moreover, the captive lifespan of released sterile males was similar to that of wild males of unknown age (Table 4 and Table 6), which indicates similar frailty dynamics and, therefore, comparable performance in the wild.

Mark–release–recapture studies are considered essential for understanding mosquito behavior, dispersal, survival, and population dynamics, particularly in the context of SIT programs. One of the key components of MRR studies is marking released males, which enables their identification upon recapture [17,30,31]. Our findings indicate no significant differences in longevity between lab-kept marked and non-marked males, suggesting that the marking process does not adversely affect survival under controlled conditions. This result aligns with previous studies demonstrating that marking techniques, such as the use of fluorescent dust, generally have a minimal impact on mosquito survival [32,33]. Therefore, our results provide additional support for the continued use of marking in field studies aimed at tracking and monitoring mosquitoes without affecting their fitness [33,34]. 

Mass production is a cornerstone of SIT programs as it allows for the large-scale rearing of sterile males, which is considered necessary to achieve area-wide population suppression. One of the most important findings of this study was the fact that males produced and sterilized in Italy (at the CAA mass production facility) and transported to Greece (BPI) exhibited significantly longer longevity compared to their non-sterile counterparts when released and recaptured. This result is particularly counterintuitive, as sterilization through irradiation or other methods is typically expected to introduce stress and consequently reduce lifespan, or at best, leave lifespan unaffected. Our findings indicate that sterilization had no negative effect on male longevity, aligning with earlier studies on *Anopheles* spp. males [35] and *Ae. albopictus* [36]. Notably, the longevity of sterile males exceeded that of non-sterile males, regardless of their previous exposure to the wild. However, our data indicate that the released males lived longer after recapture and transfer to the laboratory than those that never experienced the wild, and the longer the time spent in the wild, the greater the longevity recorded in the laboratory. This suggests that the experience of the wild may enhance post-capture lifespan, as has been documented in other mosquito species and other Diptera [18,21,22]. Although various factors may influence frailty dynamics in the wild, it is not expected that the rather short exposure of released males would increase lifespan. Beneficial effects may arise, among other things, from experiencing fluctuating conditions in the wild, resulting in a positive acclimatization effect, as well as from feeding on variable and apparently richer food sources. Besides the beneficial effects of wild exposure, demographic selection may also account for the observed patterns in longevity. Indeed, the small proportion of recaptured males may consist of the most robust individuals that exhibit longer lifespans in captivity as well. Additional research needs to be conducted to provide answers to the questions above. 

Similar to previous studies highlighting the negative effects of mass rearing and transportation on the performance of sterile males, including longevity [14,37,38,39], our study reveals slight but significant differences in longevity between the two different production sites when transportation is involved. Specifically, males produced in Greece (small-scale production, not transported) exhibited significantly longer lifespans than their Italian counterparts (mass production, transported). Conditions experienced during transportation may be detrimental for males [14]. On the other hand, crowding during both adult and larval rearing in large-scale mass rearing facilities, together with the handling of insects, may deteriorate the quality of produced males and reduce longevity.

The observed increase in the longevity of recaptured sterile males compared to non-sterile ones, which may be related to possible positive effects of sterilization, was unexpected as well. The beneficial effects of sterilization may be related to hormetic effects (where a low dose of stressor can trigger positive effects) or differential demographic selection between sterile and non-sterile males. Because of similar recapture rates between sterile and non-sterile males, the differential selection explanation is a rather weak argument. The significant interaction between release and sterilization that we found in the current study deserves to be further explored with a more targeted experimental protocol. Whether sterilization extends lifespan in male mosquitoes under the variable environment of the wild is an interesting outcome of the current study that needs further investigation to clarify the role of hormesis [40]. The inclusion of pre-release treatments and environmental stressors on the longevity of sterile males should also be addressed [36,41]. 

It is important to note a limitation regarding the males produced in Greece (BPI small-scale lab), as they were not released, sterilized, transported, or exposed to the external environment. In this study, the comparison was made exclusively between sterile and non-sterile males from Italy (mass production facility). Similarly, for the Italian males, certain variables, such as the effects of transport and marking, could not be fully analyzed, as all males from this origin were both transported and marked. Variations in laboratory rearing conditions, nutrition, irradiation, and transportation between the two production sites, as explained above, may have also contributed to differences in lifespan, highlighting the importance of optimizing mass-rearing practices in SIT programs.

Another limitation of the current study is that the comparison between lab-kept males and field-recaptured males does not reflect true mortality rates, since the longevity data for recaptured males only represents those that were able to survive post-release. This survival bias makes it difficult to fully assess the actual mortality rates of released males in the field.

In conclusion, this is the first study to apply the CCM to study the frailty dynamics and field performance of sterile, released *Ae. albopictus* males. Our results revealed the unexpected finding that sterile males, produced and transported, exhibited longer longevity than their non-sterile counterparts, especially after being released and recaptured from the wild. This result points to the possibility of a hormesis effect, where irradiation may enhance survival. Overall, these findings emphasize the need for further investigation into optimizing rearing, sterilization, and transportation methods in SIT programs to enhance the performance and longevity of sterile males in the field.

## Figures and Tables

**Table 1:** Details of the *Aedes albopictus* male categories considered in the current study. The effects of the following factors were tested: marking (marked vs. non-marked), sterilization (sterile vs. non-sterile), release status (released vs. non-released), and production origin (Centro Agricoltura Ambiente (CAA) vs. Benaki Phytopathological Institute (BPI)).

No.	Treatment Status	Description
1	Control 1 (BPI lab, marked)	Males produced in BPI (small-scale production); marked with fluorescent dye
2	Control 2 (BPI lab, non-marked)	Males produced in BPI (small-scale production); non-marked
3	Control 3 (CAA, sterile)	Males produced in CAA (mass production), irradiated at 35 Gy, transferred from Italy to Greece; not released
4	Control 4 (CAA, non-sterile)	Males produced in CAA (mass production), transferred from Italy to Greece; not released
5	Control 5 (non-treated area)	Wild males collected in Artemida (untreated area)
6	CAA (sterile males)	Males produced in CAA (mass production); irradiated at 35 Gy; transferred from Italy to Greece; released in Vravrona (treated area)
7	CAA (non-sterile males)	Males produced in CAA (mass production), transferred from Italy to Greece; released in Vravrona (treated area)
8	Wild males	Wild males were caught using the Human Landing Catch (HLC) technique in the areas of Vravrona and Artemida

**Table 2:** The effect of marking on the average longevity of males obtained from a small-scale production under laboratory conditions. Males were produced using small-scale production (BPI).

Treatment	N	Average Lifespan in Days ± SD (Min–Max Days Lived) *
Marked individuals	50	29.04 ± 16.56 ^a^ (11–72)
Non-marked individuals	50	33.44 ± 20.83 ^a^ (4–72)

* Different superscripts indicate significant differences according to the Wilcoxon Mann–Whitney test (*p* < 0.050).

**Table 3:** Mass production of sterile vs. non-sterile males transported and kept in the laboratory. Key statistics for the sterile vs. non-sterile groups included minimum, maximum, and average lifespan. Males were produced in a mass production facility (CAA), transported to Greece (BPI), and kept in the laboratory.

Treatment Status	N	Average Lifespan in Days ± SD (Min–Max Days Lived) *
Sterile males	100	23.70 ± 14.71 ^a^ (5–103)
Non-sterile males	101	23.25 ± 13.16 ^a^ (3–75)

* Different superscripts indicate significant differences according to the Wilcoxon Mann–Whitney test (*p* < 0.050).

**Table 4:** The effect of sterilization on field performance in the mass production of sterile vs. non-sterile males that were transported and released. Key statistics for the sterile vs. non-sterile groups included minimum, maximum, and average lifespan. Males were produced in a mass production facility (CAA), transported to Greece (BPI), and released.

Treatment Status	N	Average Lifespan in Days ± SD (Min–Max Days Lived) *
Sterile males (1st Release)	56	35.66 ± 21.08 ^a^ (7–112)
Non-sterile males (1st Release)	49	20.48 ± 10.63 ^b^ (6–47)
Sterile males (2nd Release)	109	34.40 ± 23.48 ^a^ (8–115)
Non-sterile males (2nd Release)	108	23.29 ± 11.42 ^b^ (7–73)

* Different superscripts indicate significant differences according to the Kruskal–Wallis test (*p* < 0.050) and post-hoc Dunn’s test with Bonferroni correction.

**Table 5:** Summary of the Cox Regression univariate analysis of the longevity of males produced in a mass production facility before transportation.

Factors	HR	95% CI	*p*-Value
Release	0.75	0.63–0.90	<0.01
Days out	0.95	0.92–0.98	<0.01
Sterilization	0.47	0.37–0.60	<0.0001
Release and Sterilization	0.46	0.32–0.66	<0.0001

**Table 6:** Key statistics for the wild males’ group included minimum, maximum, and average lifespan (Vravrona: treated area; Artemida: non-treated area).

Treatment Status *	N	Average Lifespan in Days ± SD (Min–Max Days Lived) **
Artemida	28	34.71 ± 18.87 ^a^ (6–72)
Vravrona	231	31.87 ± 21.03 ^a^ (1–107)

* In the case of wild males captured in the localities of Artemida and Vravrona, the reference is made to post-capture longevity and not to complete lifespan. ** Different superscripts indicate significant differences according to the Kruskal–Wallis test (*p* < 0.050) and post-hoc Dunn’s test with Bonferroni correction.

## Data Availability

All data are available in the manuscript.
